# Hypercohones D–G, New Polycyclic Polyprenylated Acylphloroglucinol Type Natural Products from *Hypericum cohaerens*

**DOI:** 10.1007/s13659-014-0007-5

**Published:** 2014-03-18

**Authors:** Jing-Jing Zhang, Xing-Wei Yang, Jun-Zeng Ma, Xia Liu, Li-Xin Yang, Sheng-Chao Yang, Gang Xu

**Affiliations:** 1 State Key Laboratory of Phytochemistry and Plant Resources in West China, Kunming Institute of Botany, Chinese Academy of Sciences, Kunming, 650201 People’s Republic of China; 2 University of Chinese Academy of Sciences, Beijing, 100049 People’s Republic of China; 3 Institute of Chinese Medicinal Materials, Yunnan Agricultural University, Kunming, 650201 People’s Republic of China

**Keywords:** Guttiferae, *Hypericum cohaerens*, Acylphloroglucinol, Hypercohones D–G

## Abstract

Four new polycyclic polyprenylated acylphloroglucinol type metabolites, hypercohones D–G (**1**–**4**), along with four known analogues (**5**–**8**), were isolated from the aerial parts of *Hypericum cohaerens*. The structures of these isolates were elucidated by extensive spectroscopic methods. The inhibitory activities of these isolates against five human cancer cell lines in vitro were also tested.

**Electronic supplementary material:**

The online version of this article (doi:10.1007/s13659-014-0007-5) contains supplementary material, which is available to authorized users.

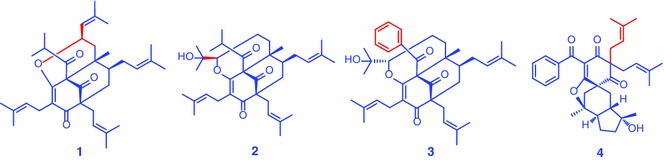

## Introduction

Polycyclic polyprenylated acylphloroglucinols (PPAPs), with a highly oxygenated and densely substituted bicyclo[3.3.1]nonane-2,4,9-trione or other related core structures decorated with prenyl or geranyl side chains, are a kind of natural products from *Hypericum*, *Clusia*, and *Garcinia* plants in family Guttiferae [1–3]. This kind of metabolites showed a wide variety of biological activities such as antitumor, antimicrobial, anti-HIV, antioxidant, and especially in the central nervous system as modulators of neurotransmitters associated with neuronal damage and depression [4–9]. Hyperforin was the most famous one in acylphloroglucinol family isolated from *H. perforatum* (St. John’s Wort) [11], and was reported to possess significant antidepressant, anticancer, and antibiotic activities [10].

The plants of genus *Hypericum*, occurring widely in temperate regions, have been used as traditional medicines in many countries all over the world [12]. *H. cohaerens* N. Robson was an endemic plant distributing in Guizhou and Yunnan provinces, P. R. China [13]. A polyprenylated acylphloroglucinol derivative with a novel bicyclo[5.3.1]-hendecane core (hypercohin A) and twelve other PPAP type natural products have been reported from this plant previously [14–16]. In our systematic search for new and bioactive PPAPs, a phytochemical investigation on the aerial parts of *H. cohaerens* was carried out and four new PPAP type metabolites (hypercohones D–G, **1**–**4**) were isolated along with four known metabolites **5**–**8**. It was notable that **4** was a spiro-cyclic type PPAP, and also was the fifth natural product with such a complex ring system. Reported herein, was the isolation, structural elucidation, and the cytotoxic bioassay of these PPAP type metabolites. 

## Results and Discussion

The MeOH extract of the air-dried and powdered aerial parts of *H. cohaerens* (10.0 kg) was subjected to a silica gel column to afford five fractions A–E. Fraction B was subjected to a series of chromatographic methods, and led to the isolation of four new acylphloroglucinol derivatives, hypercohones D–G (**1**–**4**), together with four known ones, uralodin A (**5**), [17] uralodin B (**6**), [18] oxepahyperforin (**7**) [19], and tomoeone E (**8**) [20].

Compound **1** was obtained as a light yellow gum. Its molecular formula C_35_H_50_O_4_ was deduced on the basis of its positive HRESIMS peak at *m*/*z* 557.3606 [M+Na]^+^ (calcd for 557.3606), indicating 11 indices of hydrogen deficiency. The IR spectrum showed 1731 and 1665 cm^−1^ due to carbonyl functionalities. The ^13^C NMR and DEPT spectra of **1** exhibited signals for 35 carbons, including twelve quaternary carbons (including three carbonyl carbons, five olefinic ones, and one oxygenated sp^2^ carbon), seven methines (including four unsaturated ones and an oxygenated one), five methylenes, and eleven methyls. These data indicated the characteristic signals for an acylphloroglucinol core with two nonconjugated carbonyl groups (*δ*_C_ 208.3 C-9; *δ*_C_ 210.7, C-10), an enolized 1,3-diketo group (*δ*_C_ 170.4, C-2; *δ*_C_ 133.9, C-3; *δ*_C_ 196.8, C-4), and two quaternary carbons at *δ*_C_ 73.9 (C-1), and *δ*_C_ 66.9 (C-5) [17–19] The ^1^H NMR spectrum of **1** showed the presence of four isoprenyl groups [*δ*_H_ 5.08 (1H, m, H-18), *δ*_H_ 5.06 (1H, m, H-23), *δ*_H_ 5.05 (1H, m, H-28), *δ*_H_ 5.18 (1H, d, *J* = 8.3 Hz, H-35)] and an isopropyl group [*δ*_H_ 2.38 (1H, m, H-11), *δ*_H_ 0.97 (3H, d, *J* = 6.6 Hz, H-12), *δ*_H_ 1.03 (3H, d, *J* = 6.6 Hz, H-13)] (Table 2). Based on these data, **1** was considered as an acylphloroglucinol derivative having four isoprenyl groups and an isopropyl group. The HMBC correlations from H-17 at *δ*_H_ 3.15 (1H, dd, *J* = 13.8, 8.4 Hz, H-17a), *δ*_H_ 3.08 (1H, dd, *J* = 13.8, 6.6 Hz, H-17b) to C-2 (*δ*_C_ 170.4), C-3 (*δ*_C_ 133.9), and C-4 (*δ*_C_ 196.8), from H-22 (*δ*_H_ 2.41, 2H, m) to C-4 (*δ*_C_ 196.8), C-5 (*δ*_C_ 66.9), C-6 (*δ*_C_ 41.71), and C-9 (*δ*_C_ 208.3), and the spin–spin system of H_2_-6/H-7/H-27/H-28 obtained from the ^1^H–^1^H COSY spectrum suggested the three isoprenyls were located at C-3, C-5, and C-7, respectively. Then, the remained isobutyryl was deduced to locate at C-1.

**Table 1:** ^13^C NMR data for compounds **1**–**3** in CD_3_OD (*δ* in ppm)

Position	**1** ^a^	**2** ^a^	**3** ^b^
1	73.9, C	77.8, C	73.3, C
2	170.4, C	167.1, C	169.0, C
3	133.9, C	134.4, C	128.5, C
4	196.8, C	199.0, C	196.0, C
5	66.9, C	66.8, C	65.9, C
6	41.71, CH_2_	43.2, CH_2_	41.6, CH_2_
7	39.5, CH	38.0, CH	38.2, CH
8	49.0, C	46.7, C	47.6, C
9	208.3, C	209.6, C	207.9, C
10	210.7, C	210.5, C	197.5, C
11	41.66, CH	42.3, CH	138.6, C
12	21.3, CH_3_	21.9, CH_3_	129.8, CH
13	20.7, CH_3_	21.6, CH_3_	129.8, CH
14			129.0, CH
15			133.7, CH
16			129.0, CH
17	23.4, CH_2_	23.8, CH_2_	23.3, CH_2_
18	122.0, CH	121.7, CH	121.8, CH
19	131.5, C	134.5, C	133.5, C
20	26.0, CH_3_	25.9, CH_3_	25.97, CH_3_
21	18.0, CH_3_	18.3, CH_3_	18.2, CH_3_
22	30.2, CH_2_	30.6, CH_2_	30.3, CH_2_
23	121.1, CH	120.8, CH	121.0, CH
24	134.9, C	135.0, C	135.5, C
25	26.2, CH_3_	26.2, CH_3_	26.2, CH_3_
26	18.2, CH_3_	18.2, CH_3_	18.3, CH_3_
27	28.9, CH_2_	27.7, CH_2_	27.9, CH_2_
28	123.4, CH	123.3, CH	123.5, CH
29	134.5, C	134.6, C	134.4, C
30	26.1, CH_3_	26.1, CH_3_	26.00, CH_3_
31	17.9, CH_3_	18.0, CH_3_	18.0, CH_3_
32	15.3, CH_3_	15.8, CH_3_	17.7, CH_3_
33	37.3, CH_2_	32.3, CH_2_	34.9, CH_2_
34	80.9, CH	22.8, CH_2_	24.6, CH_2_
35	125.2, CH	88.3, CH	88.2, CH
36	138.7, C	73.4, C	72.3, C
37	25.8, CH_3_	23.7, CH_3_	27.5, CH_3_
38	18.6, CH_3_	28.2, CH_3_	22.7, CH_3_

^a^Recorded at 150 MHz

^b^Recorded at 100 MHz

**Fig. 1:**
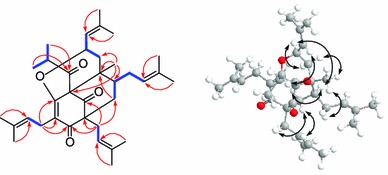
Key HMBC (), ^1^H-^1^H COSY (), and ROESY () correlations of **1**. (Color figure online)

Further analysis of the NMR data of **1** with those of hyperforin revealed that they were structurally similar to each other except that the signals for the methylene at C-34 in hyperforin was replaced by an oxygenated methine (*δ*_C_ 80.9) in **1**, [10, 11] as evidenced by the H_2_-33/H-34/H-35 unit observed in the ^1^H–^1^H COSY spectrum (Fig. 1). The existence of the epoxy group between C-34 and C-2 (*δ*_C_ 170.4) can be fully confirmed by the 11 indices of hydrogen deficiency. In addition, the HMBC correlations from Me-32 (*δ*_H_ 1.08, 3H, s) to C-1 (*δ*_C_ 73.9), C-7 (*δ*_C_ 39.5), C-8 (*δ*_C_ 49.0), and C-33 (*δ*_C_ 37.3) confirmed the structures furthermore.

The relative configuration of compound **1** was determined on the basis of a ROESY experiment (Fig. 1). The ROESY correlations of Me-32/H-27, Me-32/H-33*β*, H-34/H-33*α*, H-34/H-7, and Me-12/H-35, indicated that H-7 and H-34 were both *α*-oriented and Me-32 was *β*-oriented. Furthermore, the relative configurations of C-1 and C-5 in **1** were elucidated to be the same with those of hyperforin, as evidenced by the ROESY correlations of H-6*β*/H-23, H-6*β*/H-22, and H-6*α*/H-7. Thus, the structure of **1** was elucidated and named as hypercohone D.

**Fig. 2:**
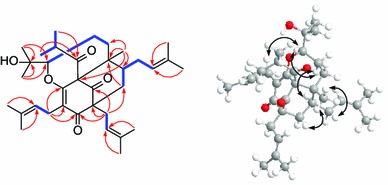
Key HMBC (), ^1^H-^1^H COSY (), and ROESY () correlations of **2**. (Color figure online)

Compound **2** was isolated as colorless oil. Based on the positive HRESIMS (*m*/*z* 553.3888 [M+H]^+^, calcd for 553.3893), the molecular formula was defined as C_35_H_52_O_5_. The IR spectrum showed absorption bands at 3441 (hydroxyl) and 1724, 1663 cm^−1^ (carbonyl groups). Extensive analysis of the 1D NMR spectroscopic data (Tables 1 and 2) of **2** exhibited a close resemblance with oxepahyperforin (**7**) [19]. The differences in the 1D spectral data of **2** compared to **7** were that the chemical shifts of C-32 (*δ*_C_ 15.8), C-33 (*δ*_C_ 32.3), C-34 (*δ*_C_ 22.8), C-35 (*δ*_C_ 88.3), and C-38 (*δ*_C_ 28.2) were all little deviated from those of **7**, which indicated that the configuration of C-35 might be differently. Carefully analysis of the ROESY spectrum revealed that H-35 was *α*-oriented, as determined by the correlations of Me-32/H-27, H-7/H-34*α*, and H-35/H-34*α*. Therefore, **2** was elucidated to be as the 35-epimer of **7** and named hypercohone E (Fig. 2).

**Table 2:** ^1^H NMR data for compounds **1**–**3** in CD_3_OD (600 MHz, *δ* in ppm, *J* in Hz)

No.	**1**	**2**	**3**
6	H*α* 1.81, dd (5.4, 13.8)	H*α* 1.88, m	H*α* 1.82, dd (4.9, 13.5)
	H*β* 1.51, dd (12.6, 13.8)	H*β* 1.41, t (13.2)	H*β* 1.69, m
7	2.18, m	1.91, m	2.10, m
11	2.38, m	2.33, m	
12	0.97, d (6.6)	1.08, d (6.6)	7.59, d (8.2)
13	1.03, d (6.6)	1.10, m	7.59, d (8.2)
14			7.31, dd (7.5, 8.2)
15			7.50, t (7.5)
16			7.31, dd (7.5, 8.2)
17	3.15, dd (8.4, 13.8)	3.18, dd (7.2, 14.4)	3.16, dd (7.2, 13.9)
	3.08, dd (6.6, 13.8)	3.12, dd (6.6, 14.4)	2.99, dd (6.8, 13.9)
18	5.08, m	5.09, t (7.2)	4.90, t (6.8)
20	1.68, m	1.67, s	1.632, s
21	1.68, s	1.68, s	1.627, s
22	2.41, m	2.44, dd (7.2, 13.8)	2.47, m
		2.37, dd (7.2, 13.8)	
23	5.06, m	4.89, t (7.8)	5.12, t (7.1)
25	1.64, s	1.59, s	1.67, s
26	1.69, s	1.66, s	1.67, s
27	H*α* 2.05, m	H*α* 1.94, m	H*α* 2.16, m
	H*β* 1.63, m	H*β* 1.62, m	H*β* 1.73, m
28	5.05, m	4.99, t (7.8)	5.02, t (7.2)
30	1.68, s	1.68, s	1.68, s
31	1.58, s	1.57, s	1.59, s
32	1.08, s	1.03, s	1.25, s
33	H*α* 1.70, m	H*α* 1.65, m	H*α* 2.06, m
	H*β* 2.21, m	H*β* 2.63, t (14.4)	H*β* 2.33, m
34	4.97, m	H*β* 2.04, m	2.02, m
		H*α* 1.77, m	1.45, m
35	5.18, d (8.3)	4.11, d (8.4)	3.73, d (9.0)
37	1.76, s	1.11, s	0.72, s
38	1.69, s	1.32, s	1.00, s

Compound **3** possessed a molecular formula of C_38_H_50_O_5_, inferred by HRESIMS at *m/z* 587.3720 [M+H]^+^ (calcd for 587.3736). Its UV absorption exhibited maximum at 278, 250, and 205 nm. The IR spectrum displayed peaks at 3440 cm^−1^ (hydroxyl), 1723 cm^−1^ (conjugated ketone) and 1653 cm^−1^ (double-bond). Comparison of the 1D and 2D NMR data indicated that the structures of **3** and **7** were similar (Tables 1 and 2) [19]. However, the signals for the isopropyl group in **7** were replaced by signals for a phenyl group in **3**, which was confirmed by HMBC correlations from both H-12 (*δ*_H_ 7.59, d, *J* = 8.2 Hz) and H-13 (*δ*_H_ 7.59, d, *J* = 8.2 Hz) to C-10 (*δ*_C_ 197.5) and the proton spin system of H-13/H-14/H-15/H-16/H-12 observed from the ^1^H–^1^H COSY spectrum. The ROESY correlations of Me-32/H-27, Me-32/H-33*β*, H-35/H-33*β*, and H-12 or H-13/H-35 deduced that **3** had the same relative configurations as **7** at C-7, C-8 and C-35 and all assigned as *β*-orientation. Ultimately, the structure of compound **3** was deduced and named as hypercohone F.

**Fig. 3:**
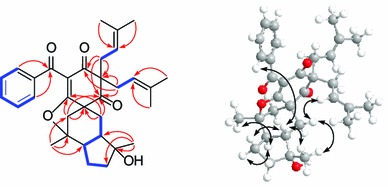
Key HMBC (), ^1^H-^1^H COSY (), and ROESY () correlations of **4**. (Color figure online)

Hypercohone G (**4**) was isolated as a light yellow gum. The molecular formula, C_33_H_40_O_5_, was determined by HREIMS (*m*/*z* 516.2871 [M]^+^, calcd for 516.2876). The IR spectrum revealed characteristic bands corresponding to the hydroxyl (3451 cm^−1^), conjugated carbonyl (1715 and 1679 cm^−1^), and double-bond (1630 cm^−1^) groups. Detailed comparison of the 1D-NMR data of **4** with those of Sampsonol C disclosed that they were closely related and shared a same spiro-cyclic skeleton, except for the signal of Me-17 in sampsonol C was replaced by an isoprenyl group in **4** [21]. This can be revealed by the presence of five carbon signals ascribable for a prenyl at *δ*_C_ 41.4 (t, C-17), 119.8 (d, C-18), 137.0 (s, C-19), 26.3 (q, C-20), and 17.8 (q, C-21) (Table 3). The mentioned isoprenyl group was located at C-6 by the HMBC correlations of H-17 to C-1 (*δ*_C_ 199.3), C-5 (*δ*_C_ 209.8), and C-6 (*δ*_C_ 65.4) coupled with the correlation of H-17/H-18 observed in ^1^H-^1^H COSY spectrum. The ROESY interactions between Me-15/H-12, H-12/H-11*α,* H-11*β*/H-8, and H-8/Me-16 suggested that **4** had the same relative configurations as Sampsonol C at C-8, C-9, C-12, and C-13 (Fig. 3). Therefore, the structure of **4** was established as illustrated and named hypercohone G.

Compounds **1**–**8** were tested for in vitro inhibitory activities against HL-60, SMMC-7721, A549, MCF-7 and SW480 human tumor cell lines using the MTT method described previously [22]. The results indicated that all the compounds were inactive with IC_50_ > 30 μM.

## Experiment Section

### General Experimental Procedures

Optical rotations were obtained with a Jasco P-1020 polarimeter. UV spectra were measured on Shimadzu UV-2401A spectraphotometer. IR spectra were detected on a Bruker Tensor-27 infrared spectrophotometer with KBr pellets. 1D and 2D NMR spectra were recorded on Bruker AV-400, and Avance III-600 MHz spectrometers with TMS as the internal standard. Chemical shifts (*δ*) were expressed in ppm with reference to the solvent signals. HRESIMS analysis and HREIMS were determined on API QSTAR time-of-flight spectrometer and on Waters Auto spec Premier P776 mass spectrometer. Semi-preparative HPLC was performed on an Agilent 1100 liquid chromatography with a Zorbax SB-C18 (9.4 mm × 25 cm) column. Column chromatography was performed on Sephadex LH-20 (GE Healthcare), Silica gel (100–200 and 200–300 mesh, Qingdao Marine Chemical Co., Ltd., Qingdao, China), and Amphichroic RP-18 gel (40–63 μm, Merck, Darmstadt, Germany) and MCI gel (75–150 μm, Mitsubishi Chemical Corporation, Tokyo, Japan). Fractions were monitored by TLC and spots were visualized by heating silica gel plates sprayed with 10 % H_2_SO_4_ in EtOH.

### Plant Material

The aerial parts of *H. cohaerens* N. Robson were collected in Daguan prefecture, Yunnan Province, China, in October 2009. The plant was identified by Dr. En-De Liu, Kunming Institute of Botany, Kunming, China. A voucher specimen was deposited with Kunming Institute of Botany with identification number 200910H01.

### Extraction and Isolation

The aerial parts of the air-dried *H. cohaerens* (10.0 kg) were powdered and percolated with MeOH at room temperature and filtered. The filtrate was evaporated in vacuo to be concentrated. The crude extract (1.5 kg) was subjected to silica gel column chromatography eluted with a petroleum ether-acetone gradient (1:0, 8:1, 4:1, 2:1, and 0:1) to produce five fractions, A–E. Fraction B (86.4 g) was separated over a MCI-gel column (MeOH-H_2_O from 8:2 to 10:0) to obtain five fractions (Fr. B1–B5). Fr. B2 (22.0 g) was isolated over an MCI gel column (MeOH-H_2_O from 85:15 to 100:0) to obtain four fractions (Fr. B2a−B2d). Fr. B2a (5.0 g) was separated on a silica gel column, eluted with petroleum ether-acetone (from 50:1 to 8:2), to yield six fractions (B2a1−B2a6). Fr. B2a2 was purified by repeated silica gel columns and semipreparative HPLC (RP-18, 93 % CH_3_CN-H_2_O) to afford **1** (25 mg), **2** (5 mg), **3** (12 mg), oxepahyperforin (**7**, 14 mg). Fr. B3 (13 g) was separated over a MCI-gel column (MeOH-H_2_O from 85:15 to 100:0) to obtain five fractions (Fr. B3a–B3e). Fr. B3b was then chromatographed on a silica gel column, eluted with petroleum ether-acetone (from 9:1 to 7:3), to yield seven fractions (Fr. B3b1–B3b7). Subfraction B3b3 (200 mg) was chromatographed by semipreparative HPLC (90 % MeOH-H_2_O) to afford three fractions (Fr. B3b3a–Fr. B3b3c). Fr. B3b3a was separated by a silica gel column, using ether-ethyl acetate (9:1) as solvent system to obtain uralodin A (**5**, 16 mg) and uralodin B (**6**, 21 mg). Fr. B3b3b and Fr. B3b3c were purified by semipreparative TLC to yield **4** (5 mg) and tomoeone F (**8**, 13 mg), respectively.

### Hypercohone D (**1**)

Light yellow gum; [*α*]_D_^24^−97.0 (*c* 0.07, MeOH); UV (MeOH) *λ*_max_ (log *ε*) 270.0 (3.69) nm; IR (KBr) *ν*_max_ 2963, 2927, 2856, 1731, 1665, 1634, 1450, 1380, 1287, 1262, 1121, 1100, 1077, 803 cm^−1^; ^1^H and ^13^C NMR data, see Tables 1 and 2; positive ESIMS *m*/*z* 557 [M+Na]^+^; positive HRESIMS *m*/*z* 557.3606 (calcd for C_35_H_50_O_4_Na [M+Na]^+^, 557.3606).

### Hypercohone E (**2**)

Colorless oil; [*α*]_D_^21^−42.8 (*c* 0.12, MeOH); UV (MeOH) *λ*_max_ (log *ε*) 260.0 (3.82) nm; IR (KBr) *ν*_max_ 3441, 2969, 2927, 2857, 1724, 1663, 1628, 1449, 1382, 1121, 1094 cm^−1^; ^1^H and ^13^C NMR data, see Tables 1 and 2; positive ESIMS *m*/*z* 575 [M+Na]^+^; positive HRESIMS *m*/*z* 553.3888 (calcd for C_35_H_53_O_5_ [M+H]^+^, 553.3893).

### Hypercohone F (**3**)

Colorless oil; [*α*]_D_^23^−240.8 (*c* 0.17, MeOH); UV (MeOH) *λ*_max_ (log *ε*) 278.2 (4.00), 250.0 (4.13) nm; IR (KBr) *ν*_max_ 3440, 2967, 2927, 1723, 1693, 1653, 1600, 1448, 1383, 1252, 1217, 1113, 1087 cm^−1^; ^1^H and ^13^C NMR data see Tables 1 and 2; positive ESIMS *m*/*z* 609 [M+Na]^+^; positive HRESIMS *m*/*z* 587.3720 (calcd for C_38_H_51_O_5_ [M+H]^+^, 587.3736).

### Hypercohone G (**4**)

Light yellow gum; [*α*]_D_^18^−56.1 (*c* 0.16, MeOH); UV (MeOH) *λ*_max_ (log *ε*) 253.0 (4.32) nm; IR (KBr) *ν*_max_ 3451, 2967, 2931, 2873, 1715, 1679, 1630, 1449, 1382, 1353, 1315, 1267, 1241, 1183, 1173, 1126, 1042, 764 cm^−1^; ^1^H and ^13^C NMR data see Table 3; positive ESIMS *m*/*z* 539 [M+Na]^+^; positive HREIMS *m*/*z* 516.2871 (calcd for C_33_H_40_O_5_ [M]^+^, 516.2876).

**Table 3:** ^1^H and ^13^C NMR data for compound **4** in CD_3_OD

Position	*δ*_C_^a^	*δ*_H_ (*J* in Hz)^b^	Position	*δ*_C_^a^	*δ*_H_^b^
1	199.3, C		17	41.4, CH_2_	2.72, dd (9.0, 13.2)
2	114.7, C				2.34, dd (7.2, 13.2)
3	179.7, C		18	119.8, CH	5.03, m
4	60.1, C		19	137.0, C	
5	209.8, C		20	26.3, CH_3_	1.70, s
6	65.4, C		21	17.8, CH_3_	1.52, s
7	34.1, CH_2_	2.08, m	22	36.6, CH_2_	2.63, d (7.5)
		1.61, m			
8	50.8, CH	1.16, m	23	121.2, CH	
9	77.3, C		24	136.7, C	5.04, m
10	40.6, CH_2_	1.71, m	25	26.4, CH_3_	1.72, s
11	23.4, CH_2_	H*α* 1.63, m	26	18.1, CH_3_	1.58, s
		H*β* 0.99, m			
12	48.6, CH	2.04, dt (6.4, 12.0)	27	194.1, C	
13	93.9, C		28	139.1, C	
14	45.0, CH_2_	2.12, m	29 (33)	130.2, CH	7.81, d (8.3)
		1.87, dd (1.9, 11.7)			
15	21.9, CH_3_	1.38, s	30 (32)	129.7, CH	7.49, t (7.5, 8.3)
16	26.6, CH_3_	1.29, s	31	134.8, CH	7.63, t (7.5)

^a^Recorded at 150 MHz

^b^Recorded at 600 MHz

### Cytotoxicity Assays

The following human tumor cell lines were used: HL-60, SMMC-7721, A-549, MCF-7, and SW-480, which were obtained from ATCC (Manassas, VA, USA). All cells were cultured in RPMI-1640 or DMEM medium (Hyclone, Logan, UT, USA), supplemented with 10 % fetal bovine serum (FBS, Hyclone) at 37 °C in a humidified atmosphere with 5 % CO_2_. Cell viability was assessed by conducting colorimetric measurements of the amount of insoluble formazan formed in living cells based on the reduction of 3-(4,5-dimethylthiazol-2-yl)-2,5-diphenyltetrazolium bromide (MTT) (Sigma, St. Louis, MO, USA). Briefly, 100 μL of adherent cells was seeded into each well of a 96-well cell culture plate and allowed to adhere for 12 h before test compound addition, while suspended cells were seeded just before test compound addition, both with an initial density of 1 × 10^5^ cells/mL in 100 μL of medium. Each tumor cell line was exposed to the test compound at various concentrations in triplicate for 48 h, with *cis*-platin and paclitaxel (Sigma) as positive control. After the incubation, MTT (100 μg) was added to each well, and the incubation continued for 4 h at 37 °C. The cells were lysed with 100 μL of 20 % SDS−50 % DMF after removal of 100 μL of medium. The optical density of the lysate was measured at 595 nm in a 96-well microtiter plate reader (Bio-Rad 680). The IC_50_ value of each compound was calculated by Reed and Muench’s method [22].

## Electronic Supplementary Material

Below is the link to the electronic supplementary material.

Supplementary material 1 (DOC 7982 kb)
