# Supplementary material for: Hypercohones D–G, New Polycyclic Polyprenylated Acylphloroglucinol Type Natural Products from Hypericum cohaerens
Source: Nat Prod Bioprospect. 2014 Mar 18;4(2):73–9. doi: 10.1007/s13659-014-0007-5 (PMC4004852; doi:10.1007/s13659-014-0007-5)

**Electronic Supplementary Material**

**Hypercohones D–G, New Polycyclic Polyprenylated Acylphloroglucinol Type Natural Products from Hypericum cohaerens**

Jing-Jing Z_HANG_, Xing-Wei Y_ANG_, Jun-Zeng M_A_, Xia L_IU_, Li-Xin Y_ANG_, Sheng-Chao Y_ANG_, and Gang X_U_

^a^ State Key Laboratory of Phytochemistry and Plant Resources in West China, Kunming Institute of Botany, Chinese Academy of Sciences, Kunming 650201, Yunnan, P. R. China,

^b^ University of Chinese Academy of Sciences, Beijing 100049, P. R. China

**Corresponding Author:**

*Gang Xu*

State Key Laboratory of Phytochemistry and Plant Resources in West China, Kunming Institute of Botany, Chinese Academy of Sciences

132# LanHei Road, Kunming, Yunnan, P. R. China, 650201

Tel: +86-871-652179717.

*e*-mail: [xugang008@mail.kib.ac.cn](mailto:xugang008@mail.kib.ac.cn)

**S1.** ^1^H NMR spectrum of **1** (methanol-d_4_, 600 MHz)

**S2**. ^13^C NMR spectrum of **1** (methanol-d_4_, 150 MHz)

**S3.** HSQC spectrum of **1**

**S4.** HMBC spectrum of **1**

**S5.** ^1^H-^1^H COSY spectrum of **1**

**S6.** ROESY spectrum of **1**

**S7.** ESI MS spectrum of **1**

**S8.** HRESI MS spectrum of **1**

**S9.** IR (KBr disk) spectrum of **1**

**S10.** UV spectrum of **1** in MeOH

**S11.** ^1^H NMR spectrum of **2** (methanol-d_4_, 600 MHz)

**S12.** ^13^C NMR spectrum of **2** (methanol-d_4_, 150 MHz)

**S13.** HSQC spectrum of **2**

**S14.** HMBC spectrum of **2**

**S15.** ^1^H-^1^H COSY spectrum of **2**

**S16.** ROESY spectrum of **2**

**S17.** ESI MS spectrum of **2**

**S18.** HRESI MS spectrum of **2**

**S19.** IR (KBr disk) spectrum of **2**

**S20.** UV spectrum of **2** in MeOH

**S21.** ^1^H NMR spectrum of **3** (methanol-d_4_, 600 MHz)

**S22.** ^13^C NMR spectrum of **3** (methanol-d_4_, 100 MHz)

**S23.** HSQC spectrum of **3**

**S24.** HMBC spectrum of **3**

**S25.** ^1^H-^1^H COSY spectrum of **3**

**S26.** ROESY spectrum of **3**

**S27.** ESI MS spectrum of **3**

**S28.** HRESI MS spectrum of **3**

**S29.** IR (KBr disk) spectrum of **3**

**S30.** UV spectrum of **3** in MeOH

**S31.** ^1^H NMR spectrum of **4** (methanol-d_4_, 600 MHz)

**S32.** ^13^C NMR spectrum of **4** (methanol-d_4_, 150 MHz)

**S33.** HSQC spectrum of **4**

**S34.** HMBC spectrum of **4**

**S35.** ^1^H-^1^H COSY spectrum of **4**

**S36.** ROESY spectrum of **4**

**S37.** ESI MS spectrum of **4**

**S38.** HREI MS spectrum of **4**

**S39.** IR (KBr disk) spectrum of **4**

**S40.** UV spectrum of **4** in MeOH

**S1.** ^1^H NMR spectrum of **1** in methanol-d_4_


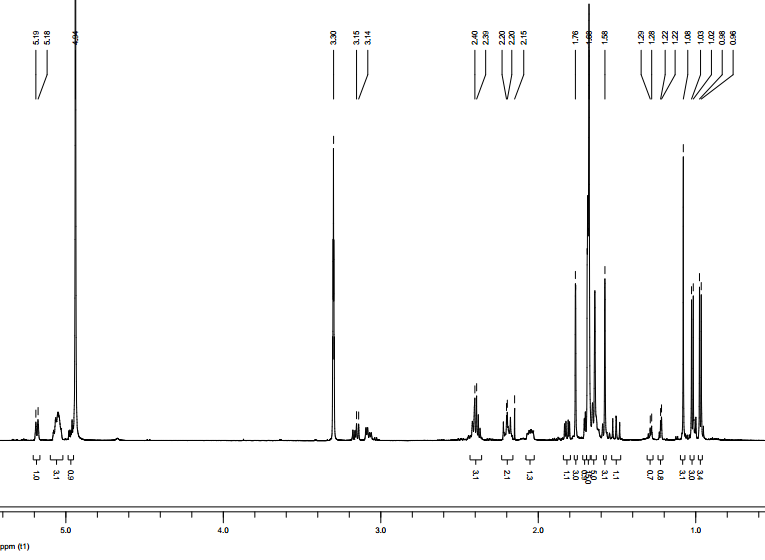


**S2.** ^13^C NMR spectrum of **1** in methanol-d_4_


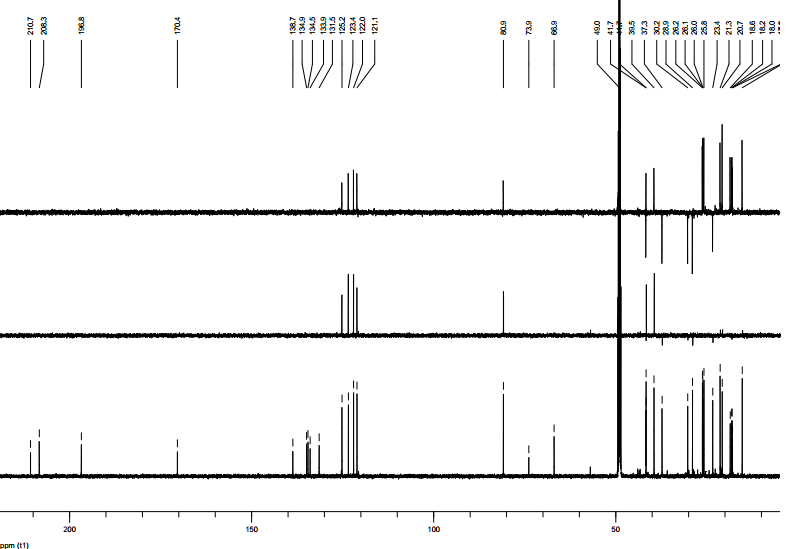


**S3.** HSQC spectrum of **1**


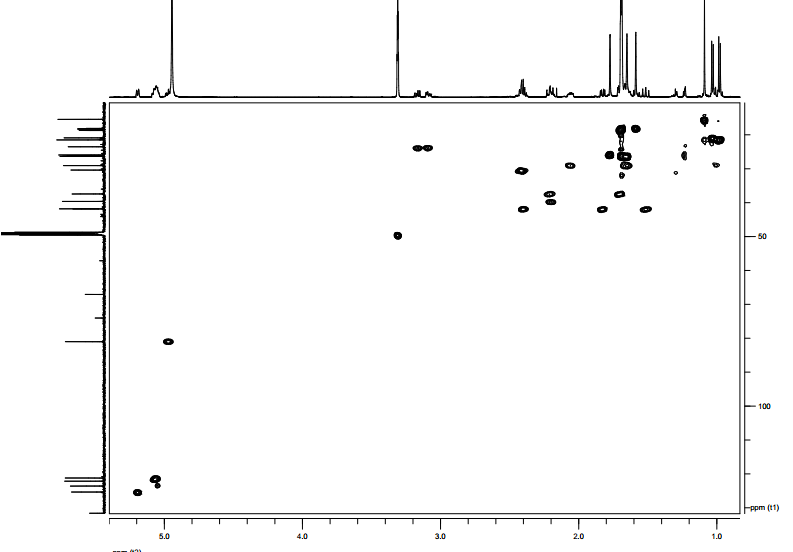


**S4.** HMBC spectrum of **1**


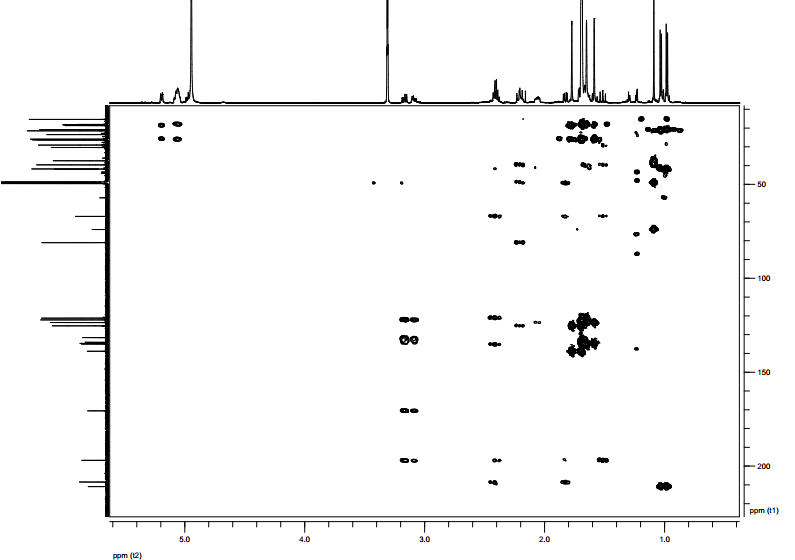


**S5.** COSY spectrum of **1**


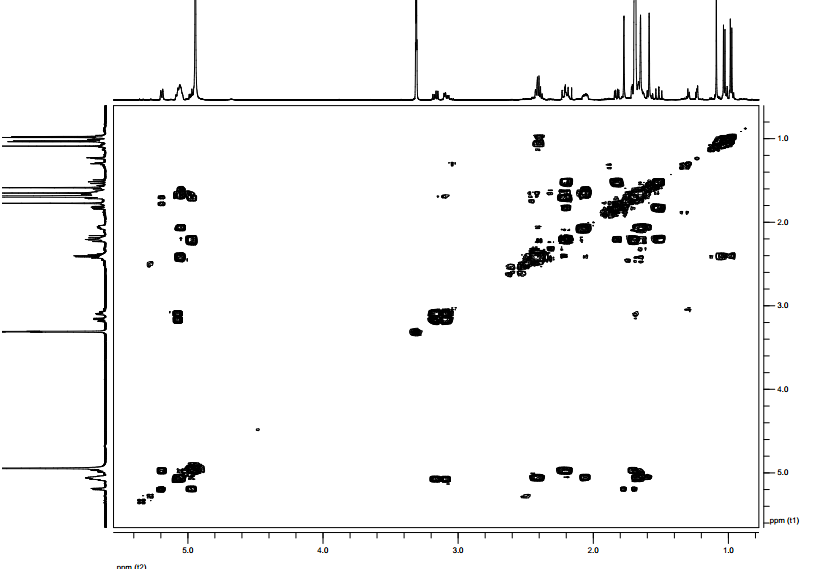


**S6.** ROESY spectrum of **1**


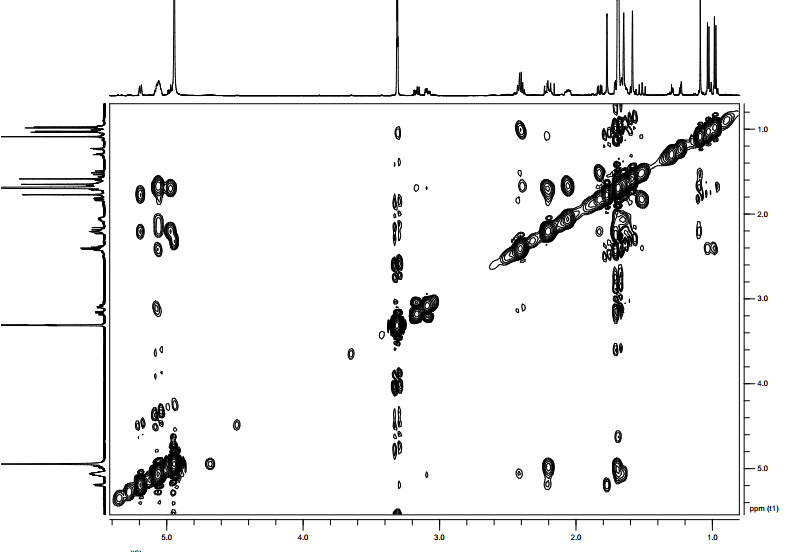


**S7.** ESI MS spectrum of **1**


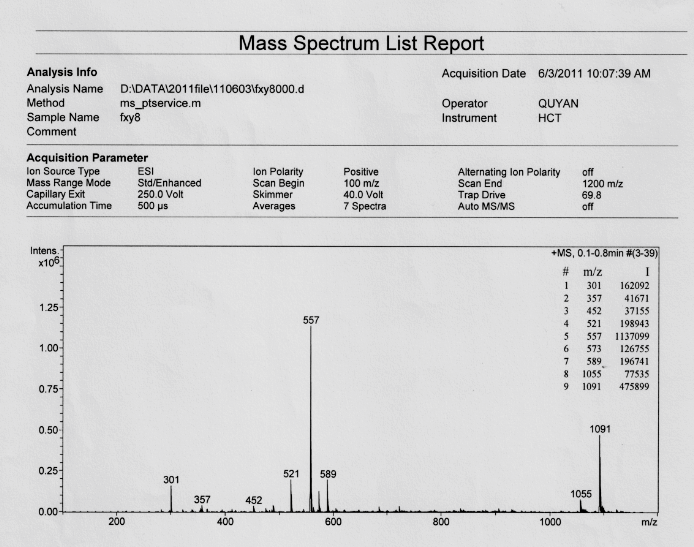


**S8.** HRESI MS spectrum of **1**


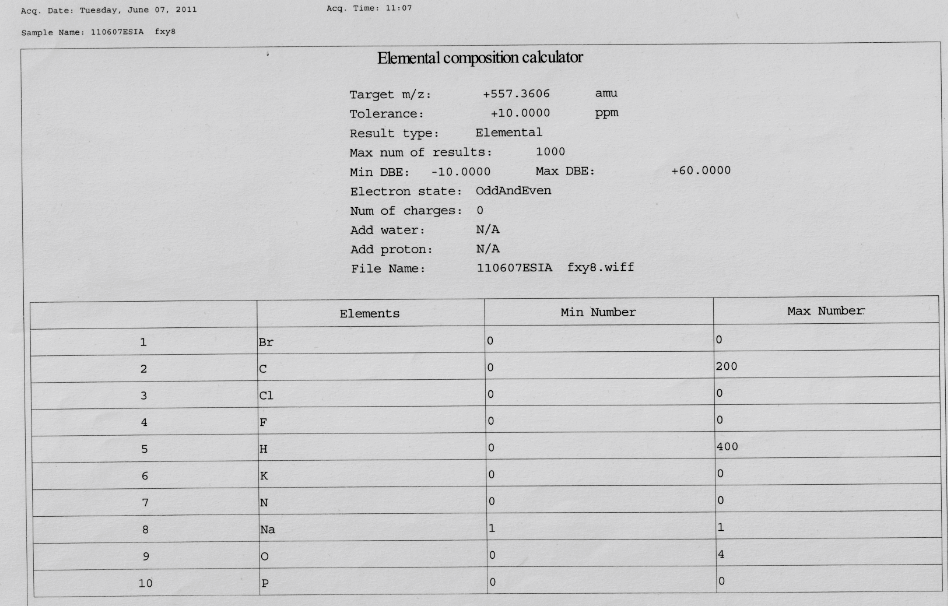


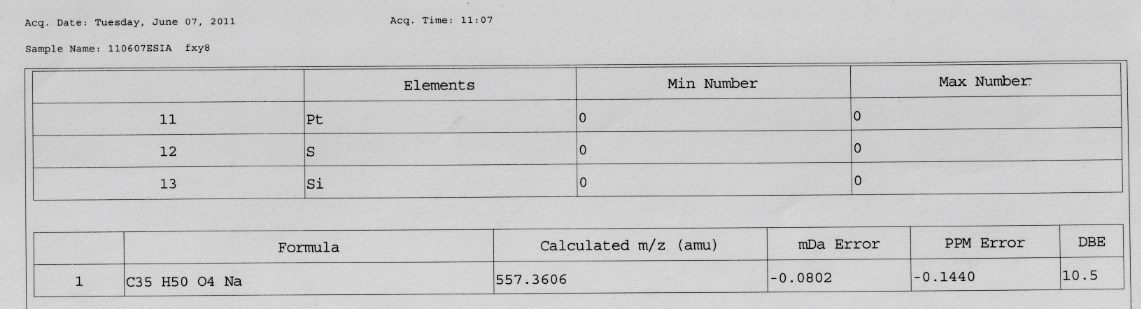


**S9.** IR (KBr disk) spectrum of **1**


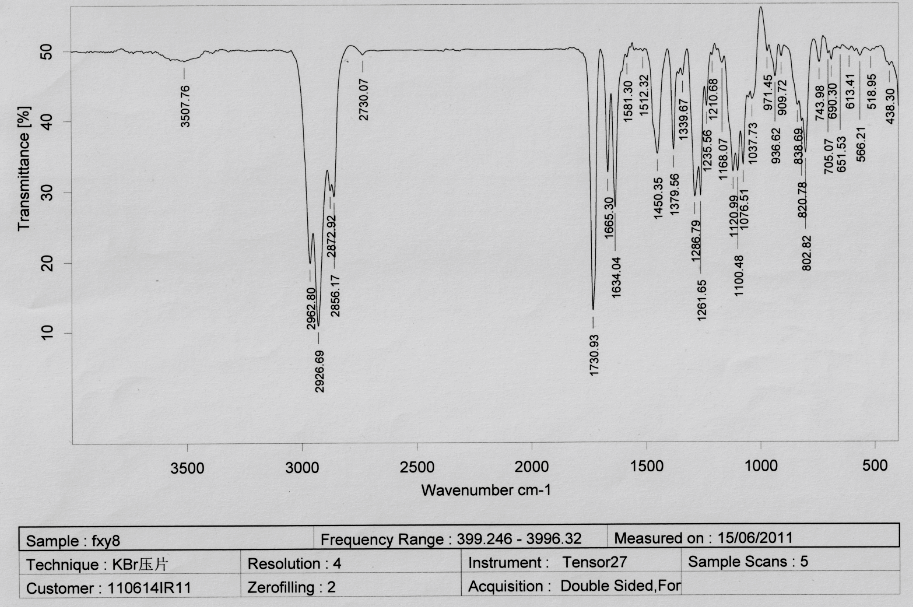


**S10.** UV spectrum of **1** in MeOH


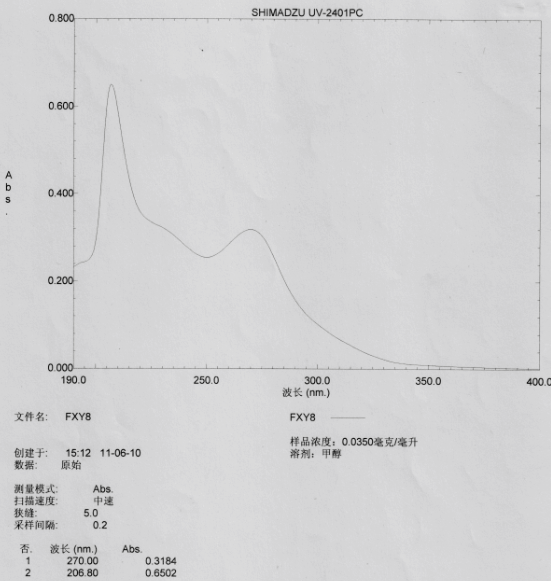


**S11.** ^1^H NMR spectrum of **2** (methanol-d_4_, 600 MHz)


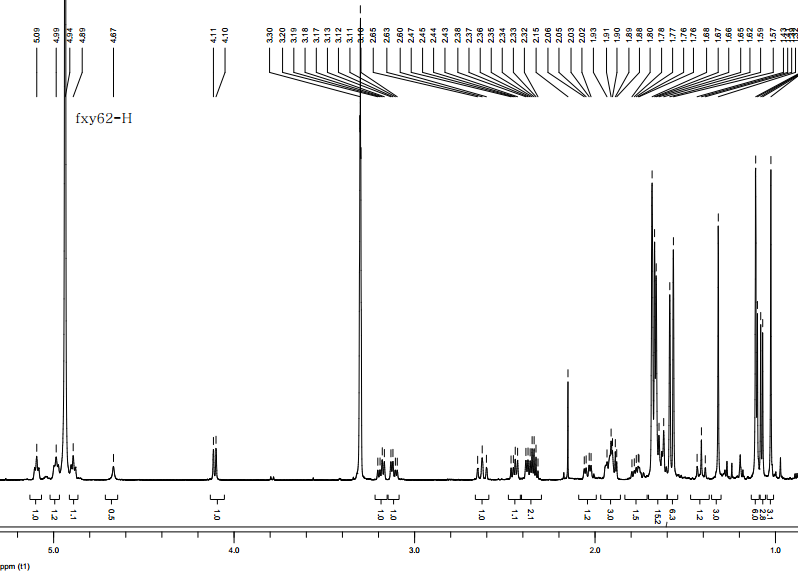


**S12.** ^13^C NMR spectrum of **2** (methanol-d_4_, 150 MHz)


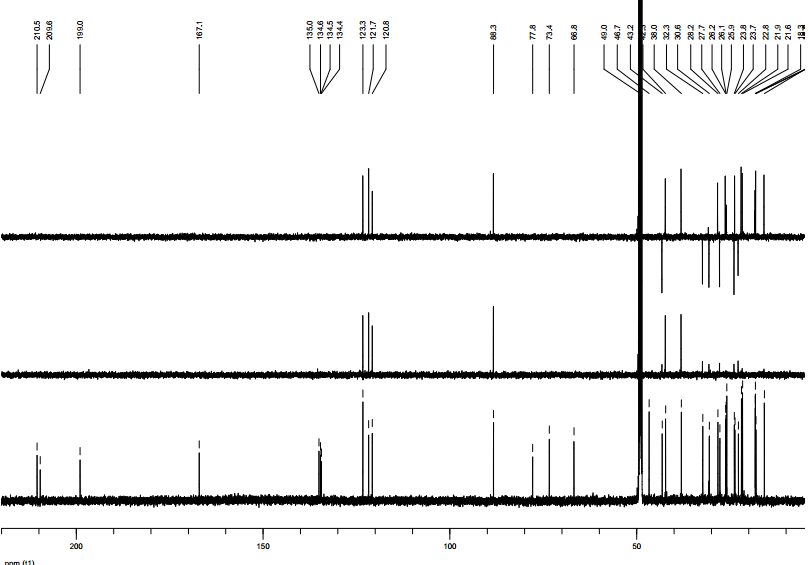


**S13.** HSQC spectrum of **2**


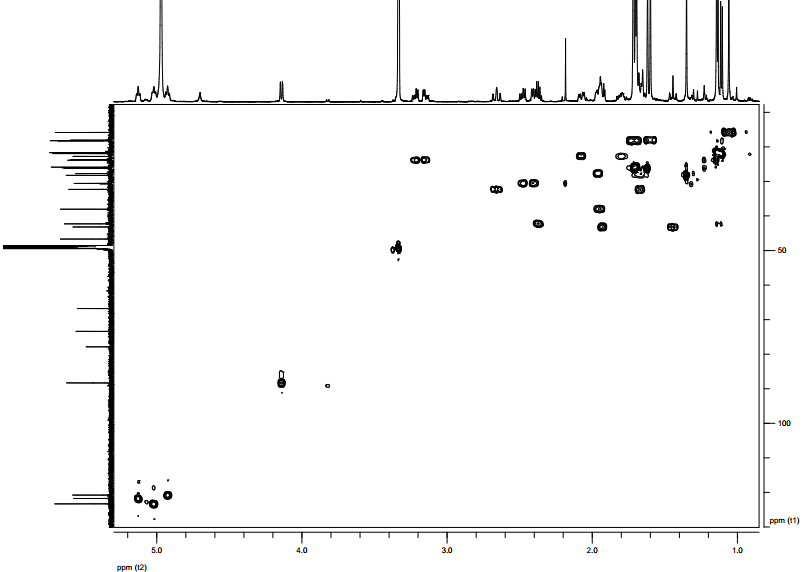


**S14.** HMBC spectrum of **2**


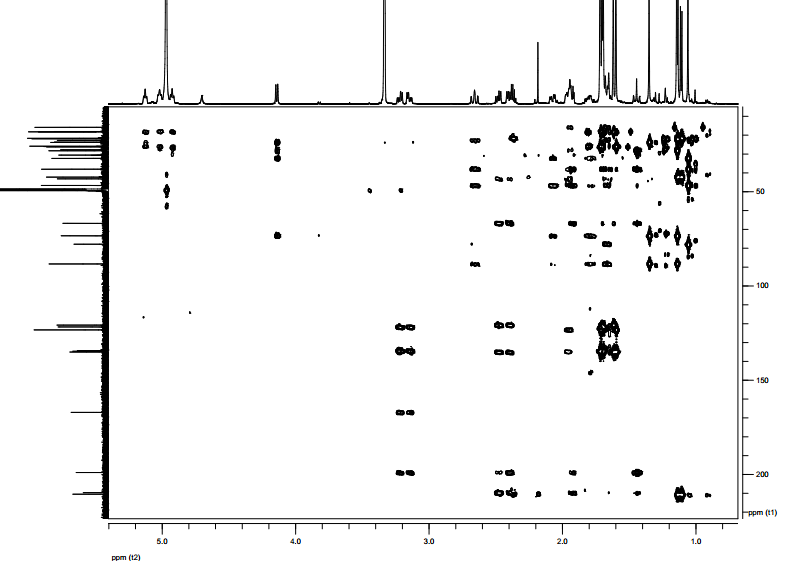


**S15.** COSY spectrum of **2**


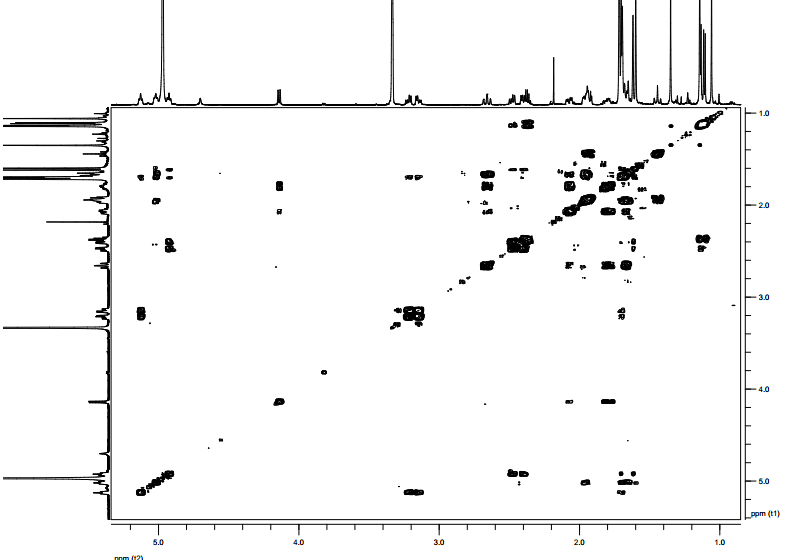


**S16.** ROESY spectrum of **2**


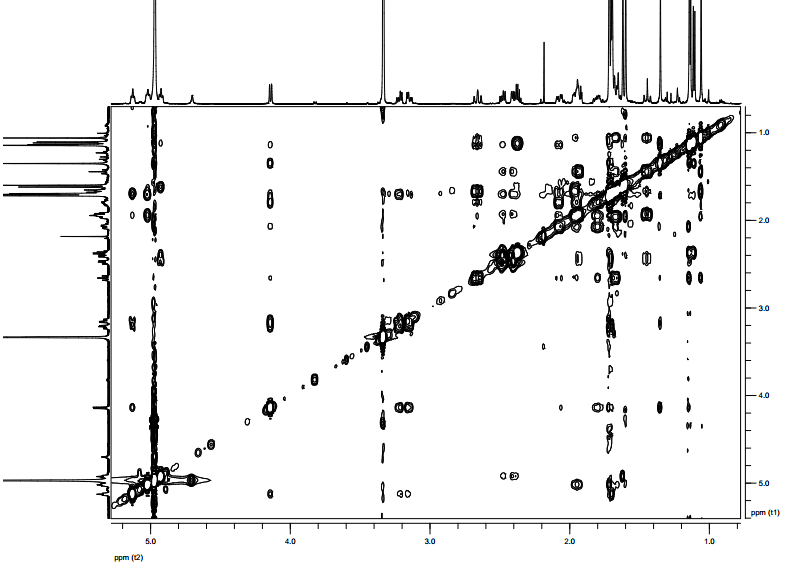


**S17.** ESI MS spectrum of **2**


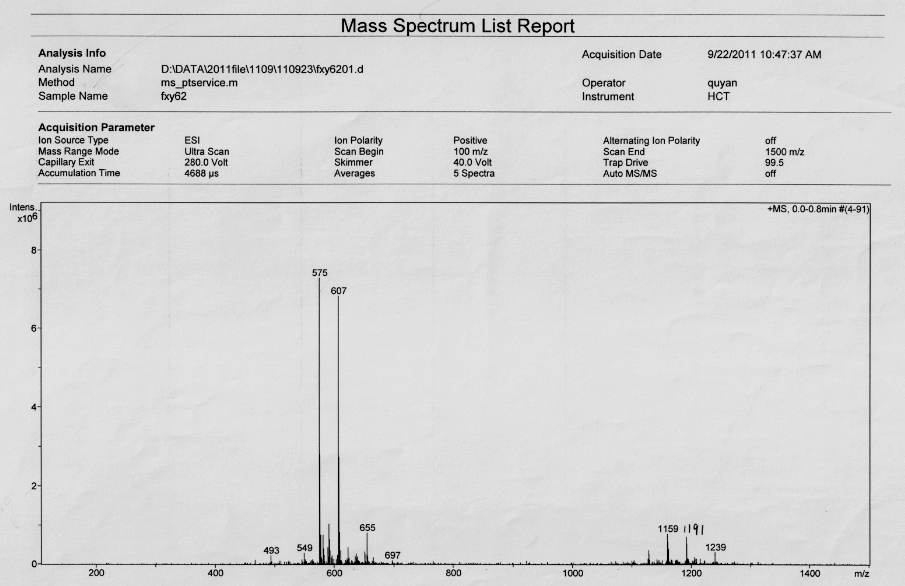


**S18.** HRESI MS spectrum of **2**


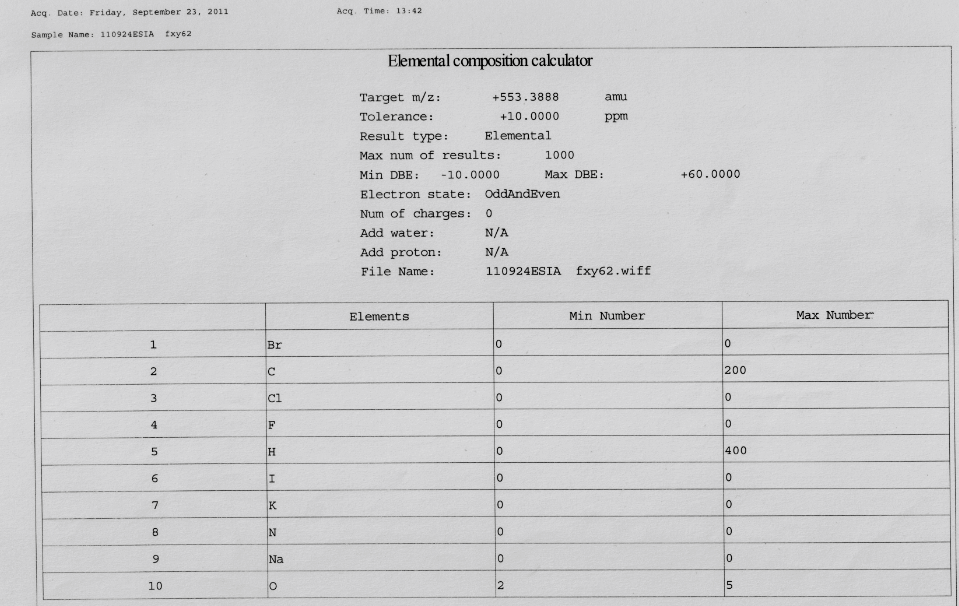


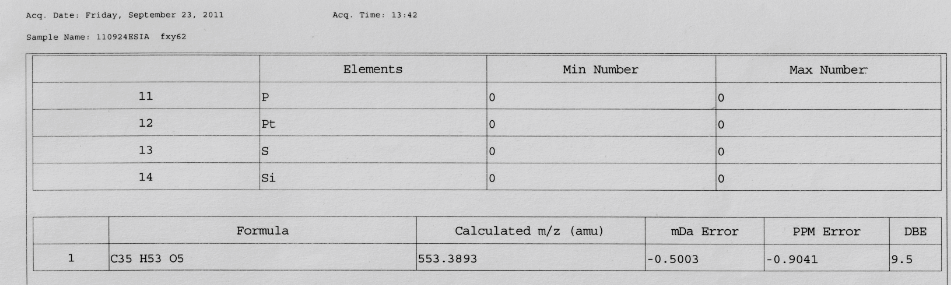


**S19.** IR (KBr disk) spectrum of **2**


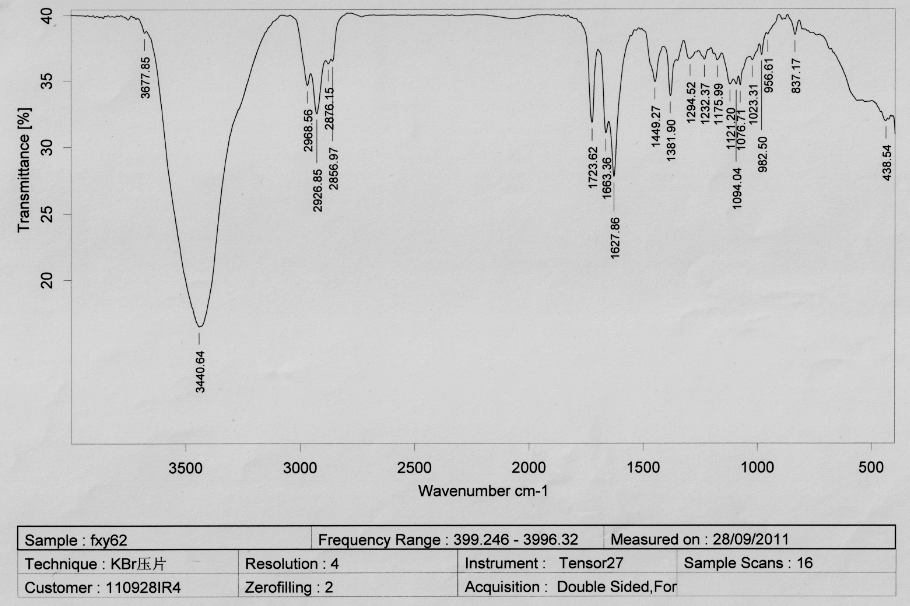


**S20.** UV spectrum of **2** in MeOH


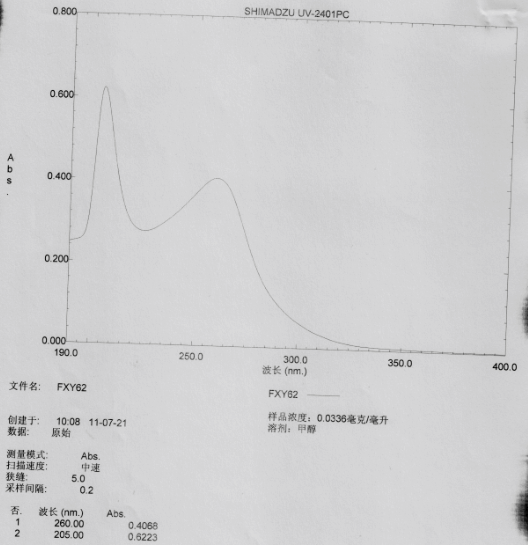


**S21.** ^1^H NMR spectrum of **3** (methanol-d_4_, 600 MHz)


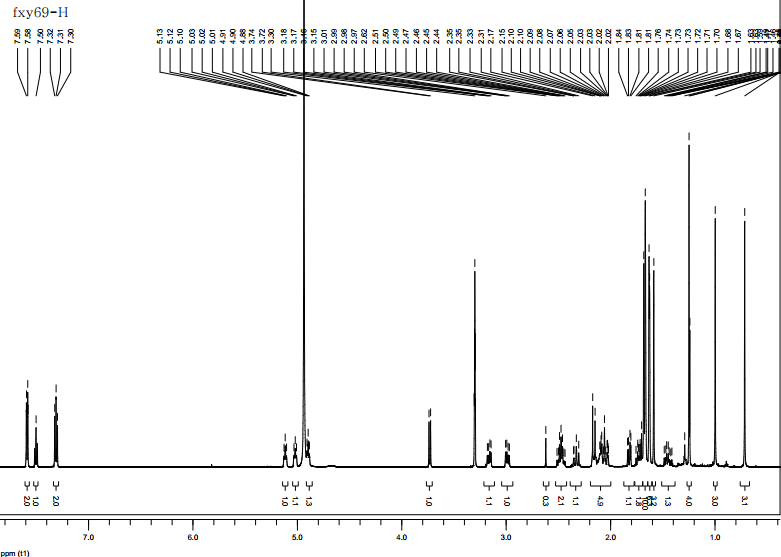


**S22.** ^13^C NMR spectrum of **3** (methanol-d_4_, 100 MHz)


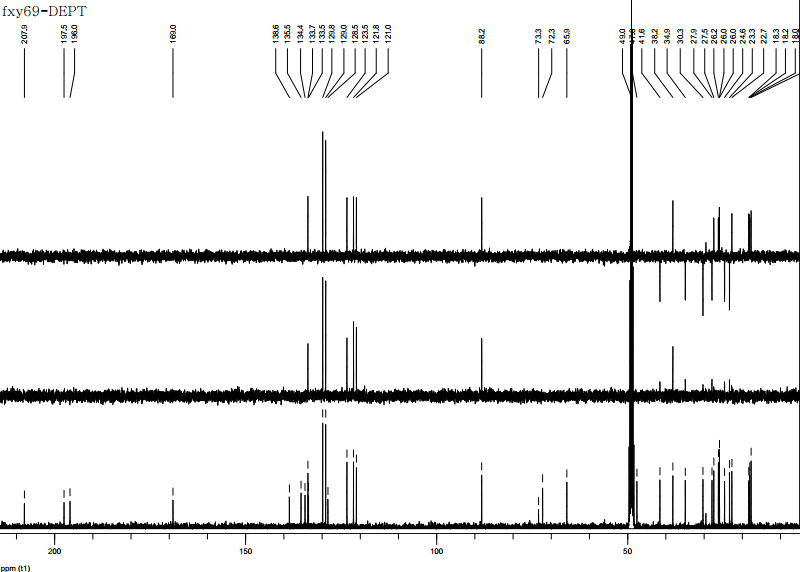


**S23.** HSQC spectrum of **3**


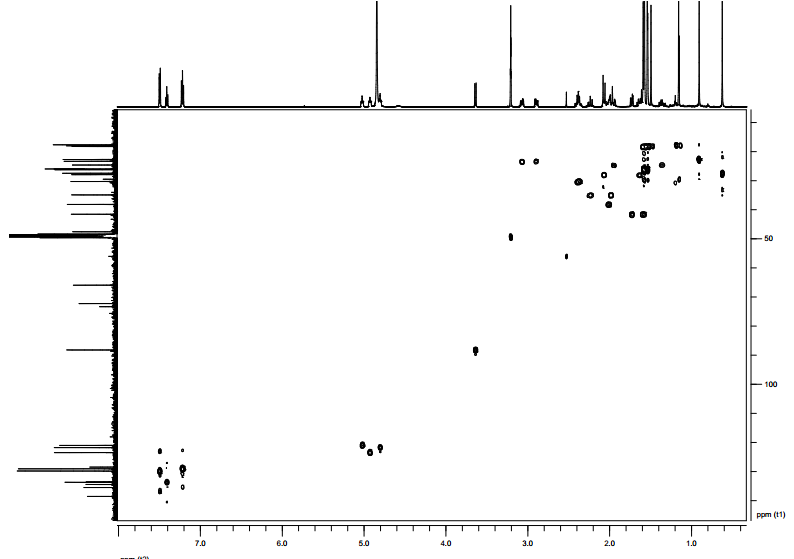


**S24.** HMBC spectrum of **3**


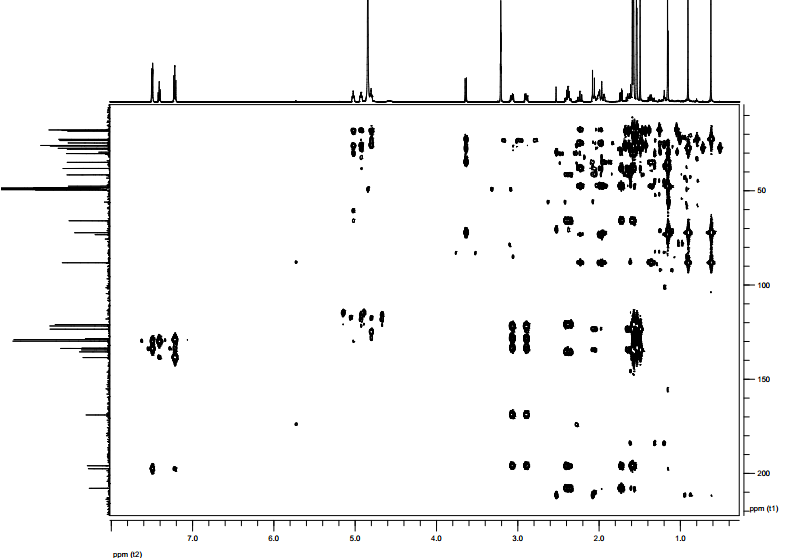


**S25.** ^1^H-^1^H COSY spectrum of **3**


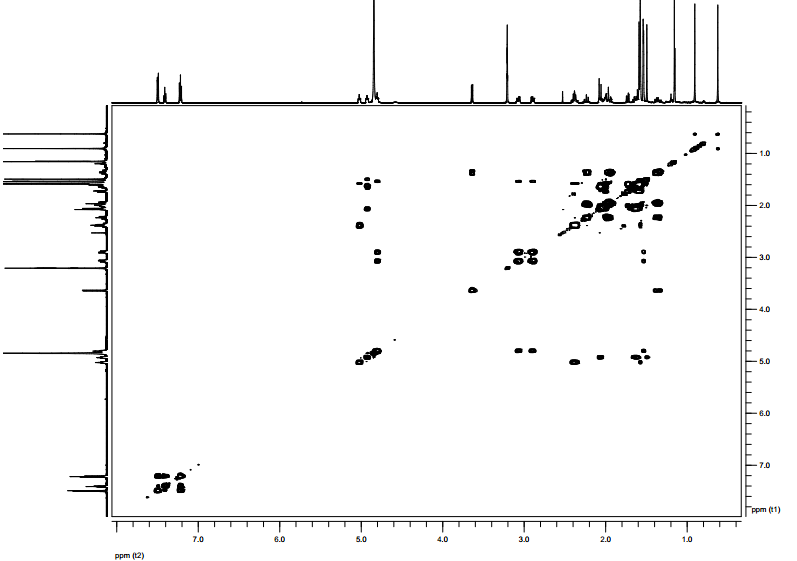


**S26.** ROESY spectrum of **3**


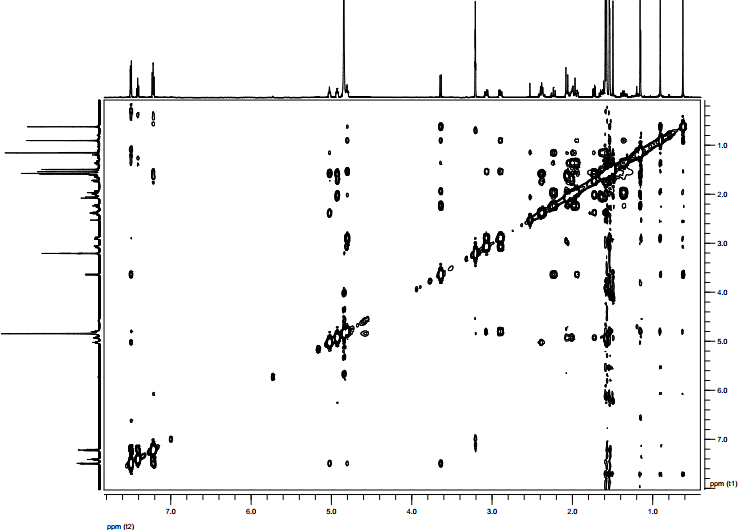


**S27.** ESI MS spectrum of **3**


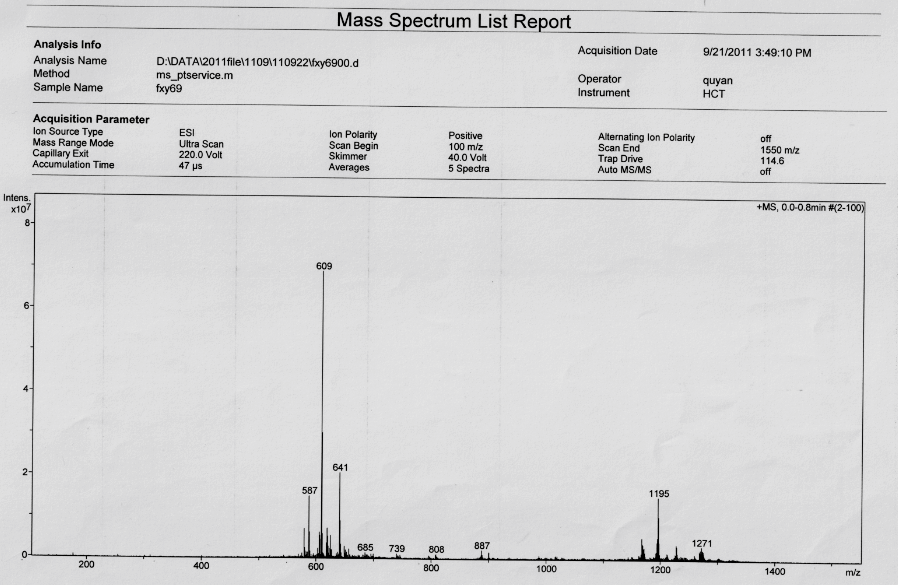


**S28.** HRESI MS spectrum of **3**


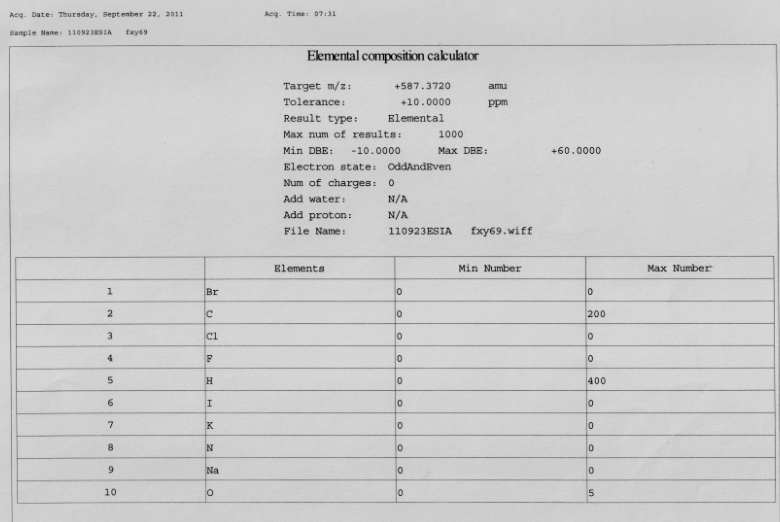


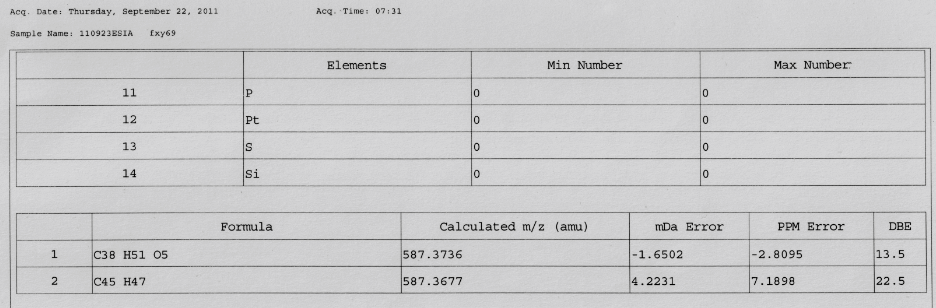


**S29.** IR (KBr disk) spectrum of **3**


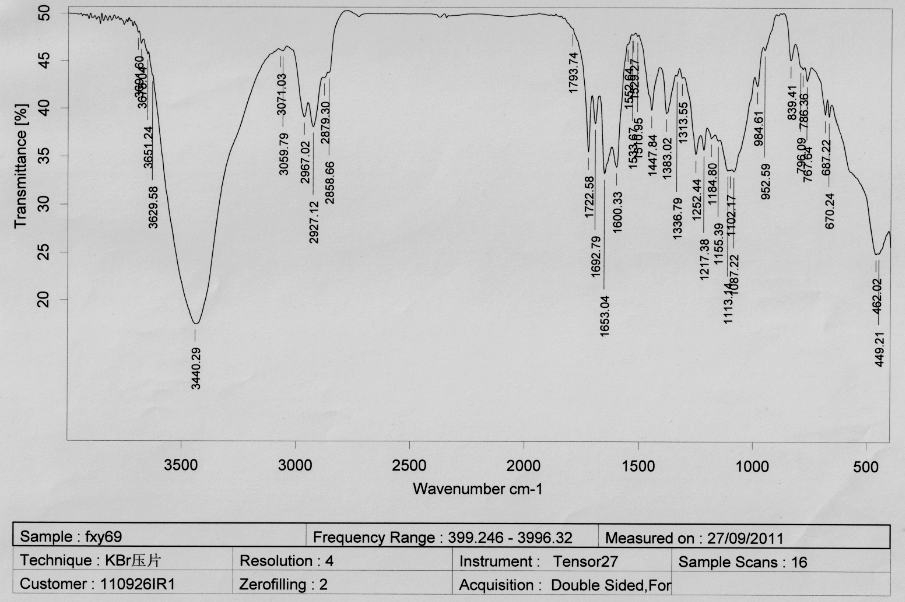


**S30.** UV spectrum of **3** in MeOH


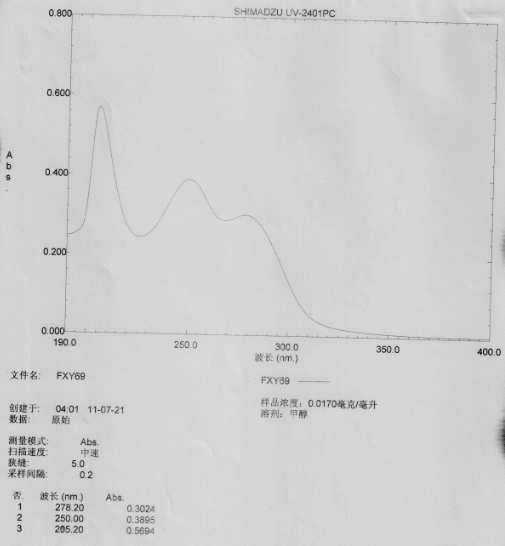


**S31.** ^1^H NMR spectrum of **4** (methanol-d_4_, 600 MHz)


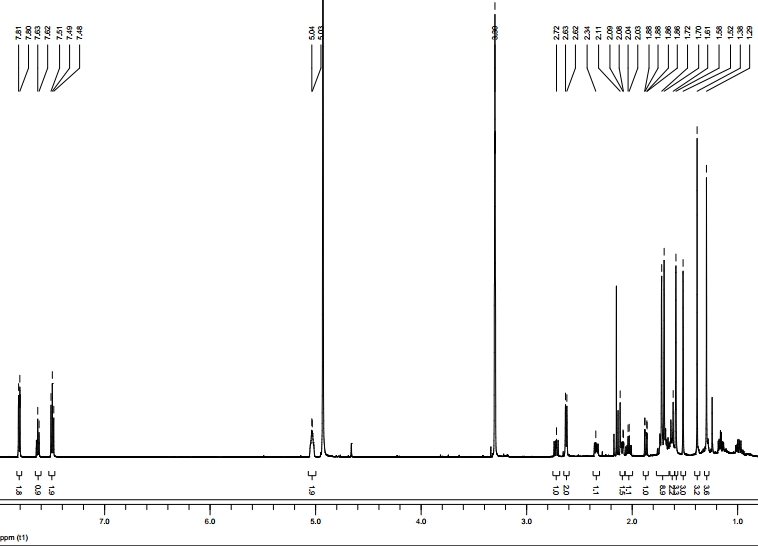


**S32.** ^13^C NMR spectrum of **4** (methanol-d_4_, 150 MHz)


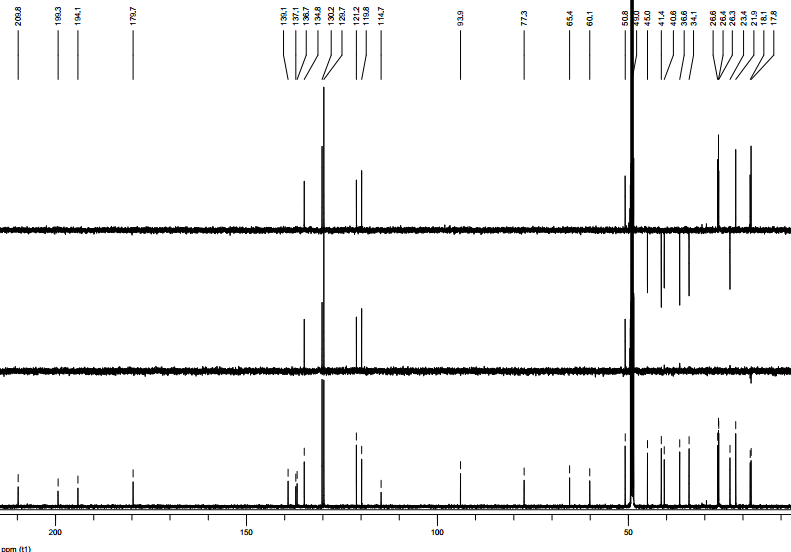


**S33.** HSQC spectrum of **4**


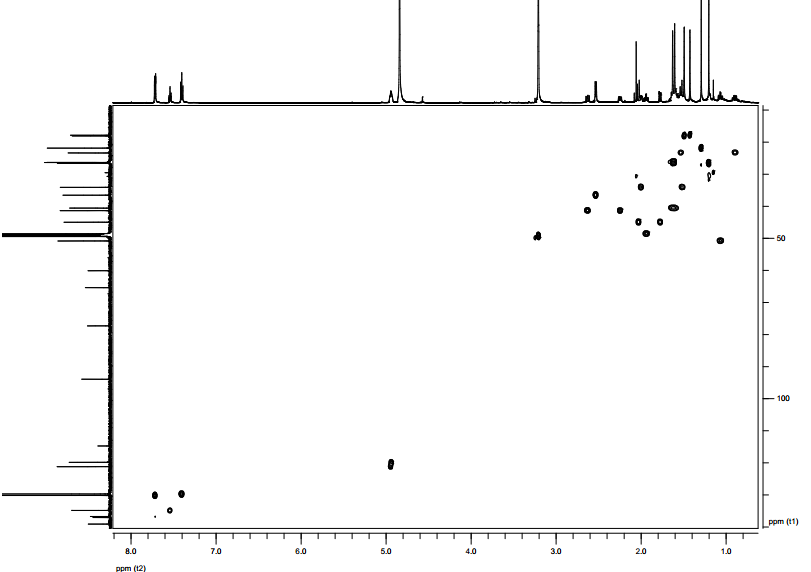


**S34.** HMBC spectrum of **4**


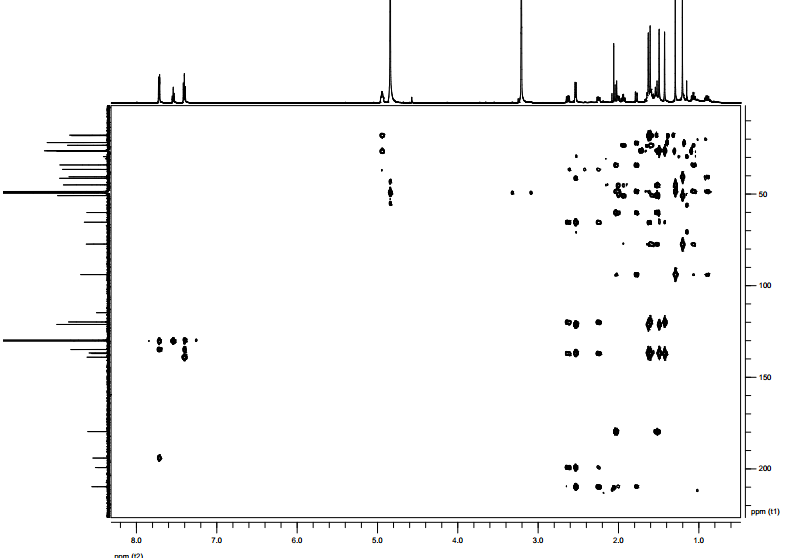


**S35.** ^1^H-^1^H COSY spectrum of **4**


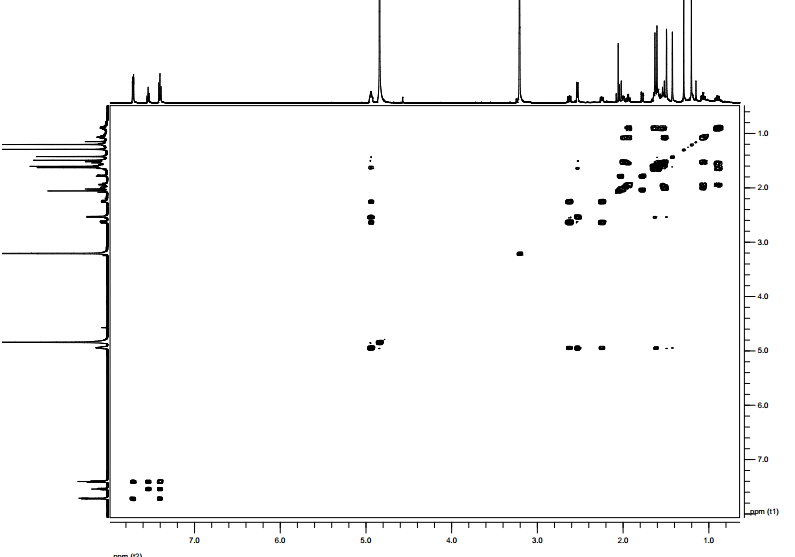


**S36.** ROESY spectrum of **4**


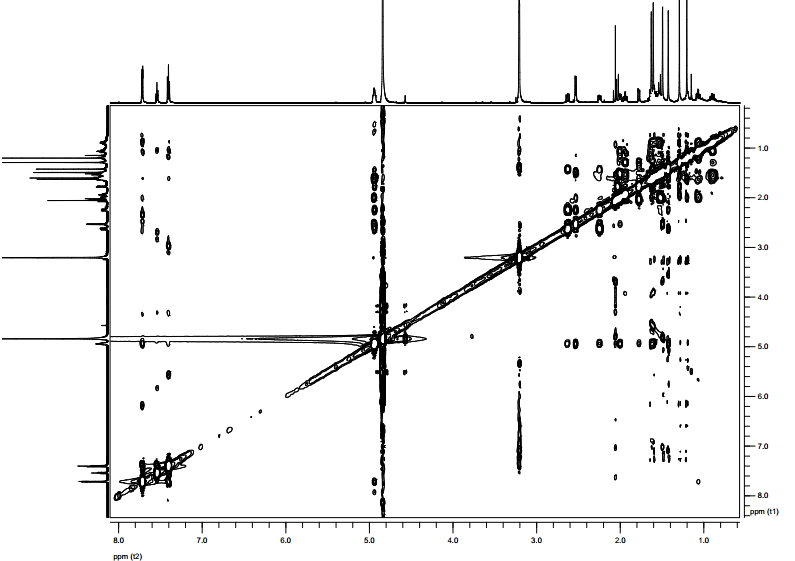


**S37.** ESI MS spectrum of **4**


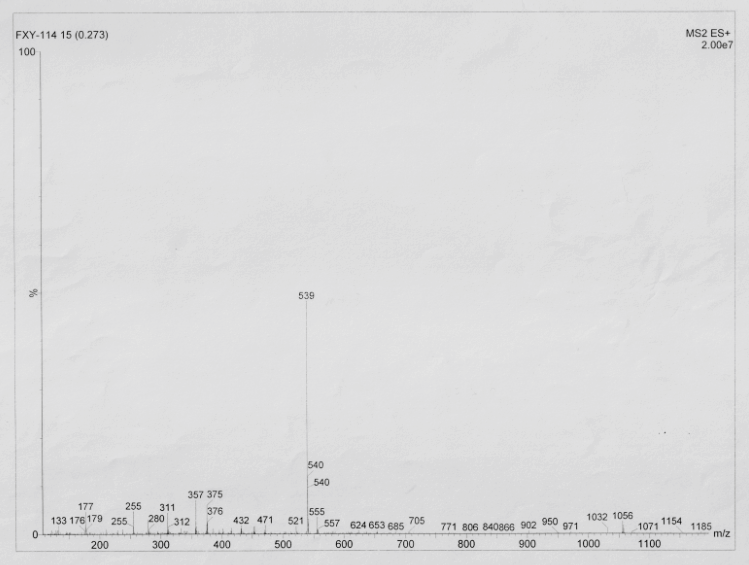


**S38.** HREI MS spectrum of **4**


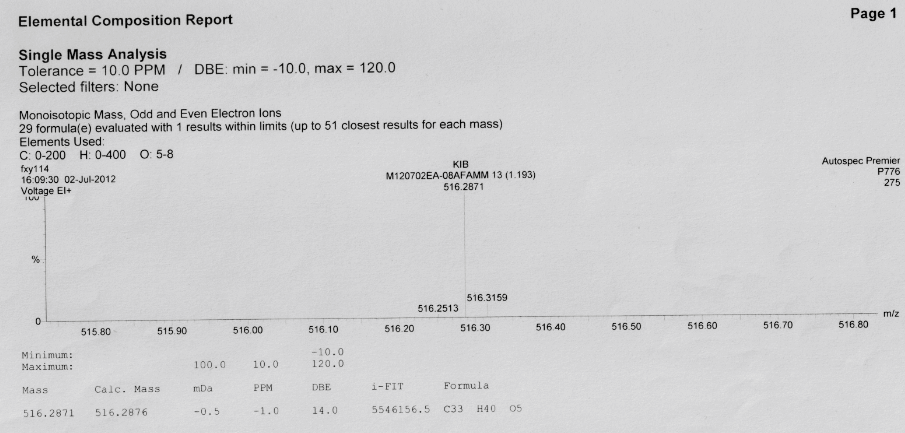


**S39.** IR (KBr disk) spectrum of **4**


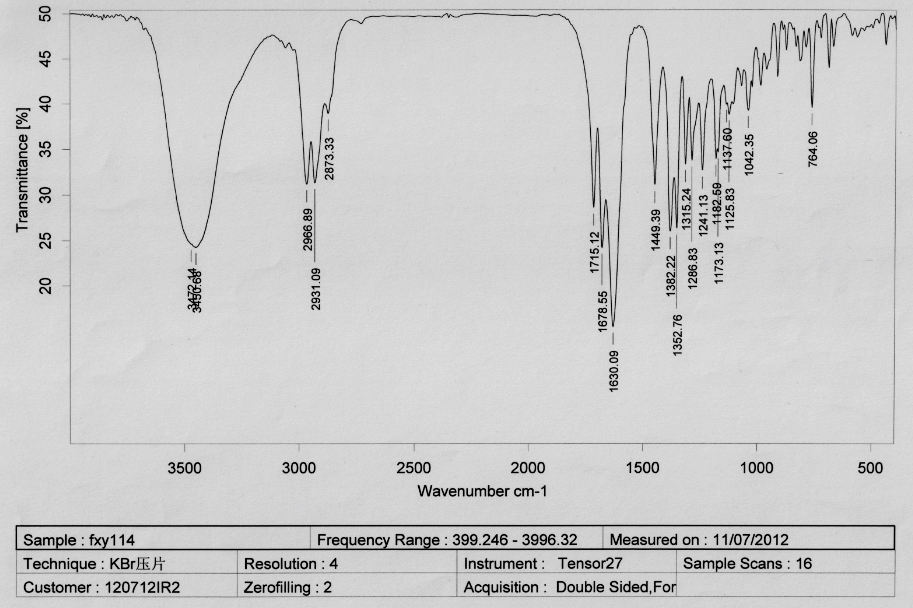


**S40.** UV spectrum of **4** in MeOH


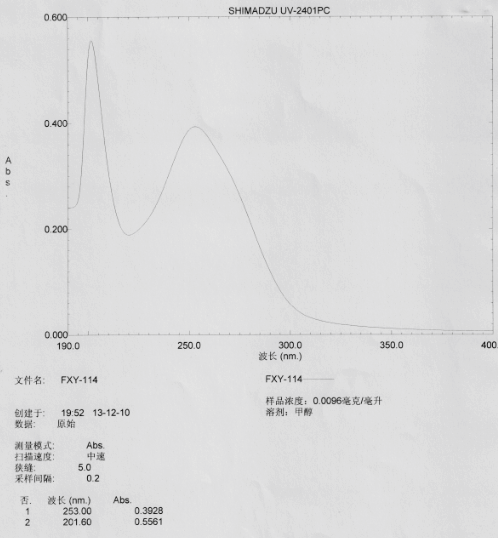

Supplement: Supplementary file 1 — Supplementary material 1 (DOC 7982 kb) [file 13659_2014_7_MOESM1_ESM.doc]
